# Supplementary material for: Resolving the full spectrum of human genome variation using Linked-Reads
Source: Genome Res. 2019 Apr;29(4):635–45. doi: 10.1101/gr.234443.118 (PMC6442396; doi:10.1101/gr.234443.118)
Supplement: Supplemental Material [file supp_gr.234443.118_Supplemental_longranger-2.2.zip › longranger-2.2.tar.bz2!/lr2.2-public/lib/stan/cmdstan/stan/doc/api/html/namespacemembers_t.html]

Stan: Namespace Members


|  |  |
| --- | --- |
|  | Stan  2.5.0  probability, sampling & optimization |


- Main Page
- Related Pages
- Namespaces
- Classes
- Files

- Namespace List
- Namespace Members

- All
- Functions
- Variables
- Typedefs
- Enumerations
- Enumerator

- a
- b
- c
- d
- e
- f
- g
- h
- i
- j
- l
- m
- n
- o
- p
- q
- r
- s
- t
- u
- v
- w


All Classes Namespaces Files Functions Variables Typedefs Enumerations Enumerator Friends Macros Pages

Here is a list of all namespace members with links to the namespace documentation for each member:

### - t -

- tail()
  : stan::math
- tan()
  : stan::agrad
- tanh()
  : stan::agrad
- tcrossprod()
  : stan::agrad
  , stan::math
- TERM\_ABSF
  : stan::optimization
- TERM\_ABSGRAD
  : stan::optimization
- TERM\_ABSX
  : stan::optimization
- TERM\_LSFAIL
  : stan::optimization
- TERM\_MAXIT
  : stan::optimization
- TERM\_RELF
  : stan::optimization
- TERM\_RELGRAD
  : stan::optimization
- TERM\_SUCCESS
  : stan::optimization
- TerminationCondition
  : stan::optimization
- test\_gradients()
  : stan::model
- tgamma()
  : stan::agrad
- to\_array\_1d()
  : stan::math
- to\_array\_2d()
  : stan::math
- to\_fvar()
  : stan::agrad
- to\_matrix()
  : stan::math
- to\_row\_vector()
  : stan::math
- to\_string()
  : stan::gm
- to\_var()
  : stan::agrad
- to\_vector()
  : stan::math
- total\_dims()
  : stan::gm
- trace()
  : stan::math
- trace\_gen\_inv\_quad\_form\_ldlt()
  : stan::agrad
  , stan::math
- trace\_gen\_quad\_form()
  : stan::agrad
  , stan::math
- trace\_inv\_quad\_form\_ldlt()
  : stan::agrad
  , stan::math
- trace\_quad\_form()
  : stan::agrad
  , stan::math
- transformed\_data\_origin
  : stan::gm
- transformed\_parameter\_origin
  : stan::gm
- transpose()
  : stan::math
- transpose\_f
  : stan::gm
- trigamma()
  : stan::math
- trunc()
  : stan::agrad
- TWO\_OVER\_SQRT\_PI
  : stan::math


---

[ Stan Home Page ]

*© 2011–2014,
Stan Development Team.*
